# Dysphagia Among Children—A Single-Center Experience

**DOI:** 10.3390/jcm14092906

**Published:** 2025-04-23

**Authors:** Katarzyna Zdanowicz, Artur Rycyk, Dariusz Marek Lebensztejn, Urszula Daniluk

**Affiliations:** Department of Pediatrics, Gastroenterology, Hepatology, Nutrition, Allergology and Pulmonology, Medical University of Bialystok, 15-274 Bialystok, Poland

**Keywords:** dysphagia, children, eosinophilic esophagitis

## Abstract

**Background/Objectives**: In recent years, there has been an increase in the prevalence of eosinophilic esophagitis (EoE), in which dysphagia is one of the main symptoms. To date, there are few data on the prevalence of EoE in pediatric patients with dysphagia. The aim of this study was to determine the causes of dysphagia in children in our region. The second aim of this study was to estimate the prevalence of EoE in children with swallowing difficulties and to see if there were differences in the characteristics of dysphagia reported by children with EoE compared to children with non-EoE-related dysphagia. **Methods**: The 6-year retrospective analysis included patients with dysphagia who were referred to our department. Children with dysphagia were further divided into two groups: group I consisted of children with dysphagia due to EoE, while group II consisted of children with dysphagia due to other causes (non-EoE). **Results**: One hundred and forty-six children between the ages 0 and 17 were enrolled into the study, including thirty-seven in group I and one hundred and nine in group II. The most common causes of dysphagia were gastrointestinal diseases, followed by neurological/psychiatric disorders. The prevalence of EoE was 25.34% in the whole study group and 41.11% considering only gastrointestinal causes of dysphagia. Children in group I were more likely to have coexisting asthma, allergic rhinitis and food allergy. There was statistically significance in higher blood eosinophil count in EoE individuals. In a multivariate binominal logistic regression model, only eosinophilia and coexisting food allergy were associated with an increased risk of EoE in patients with dysphagia. **Conclusions**: In our study, the most common cause of dysphagia was gastroenterological diseases, especially EoE. Patients with dysphagia, comorbid allergy and peripheral blood eosinophilia should be suspected for having EoE and referred for endoscopy.

## 1. Introduction

Dysphagia is a professional term to describe difficult or abnormal swallowing of food, potentially leading to serious complications and for this reason regarded as one of the alarming symptoms/red flags in gastroenterology [1]. In recent years, this condition has continued to grow among children. Based on data in the literature, the incidence of swallowing dysfunction in the pediatric population is estimated to be about 1% [2,3]. However, in the risk population, the incidence of this symptom is higher; it can occur in up to 99% of children with cerebral palsy and 10% in premature infants [4]. In addition to neurological causes and prematurity, dysphagia may occur due to structural abnormalities of the nose, oral cavity and larynx, as well as gastrointestinal (GI) or cardiopulmonary disorders. Symptoms occurring in patients depend on the location of pathology and require a different therapeutic approach. In many cases, a thorough analysis of the medical history makes it possible to initially distinguish between different types of dysphagia and carry out the appropriate diagnosis and treatment [5,6]. It is worth noting that dysphagia in adults can be associated with undernutrition [7]. Malnutrition in children in the course of dysphagia has not yet been well characterized. However, it is known that malnutrition can lead to the deterioration of children’s daily functioning, as well as the course of various diseases [8,9].

Dysphagia in adults is often the symptom of eosinophilic esophagitis (EoE) [10,11]. EoE is a chronic immune-mediated inflammatory disease with a steadily increasing incidence [12]. The diagnosis of EoE is made on the basis of clinical symptoms reflecting esophageal dysfunction with esophageal mucosa eosinophilia [13]. The single available piece of data reporting the incidence of EoE in patients with dysphagia at 10–15% applies only to adults [14,15]. It was noticed that younger age significantly increased the risk of EoE diagnosis in patients with dysphagia [14]. There is a lack of studies on this topic among children. In our previous study on children with esophagitis, we showed that dysphagia was the only symptom more frequently observed in children with EoE than in patients with other type of esophagitis [16]. In the pediatric population, symptoms of EoE depend on the age of onset and consist of gastroesophageal reflux-like symptoms, food refusal, failure to thrive in younger age groups, abdominal pain and food impaction in schoolchildren and adolescents [17]. Swallowing difficulties seem to be a significant problem reported by patients in the daily practice of general practitioners, pediatricians and gastroenterologists. Therefore, we decided to investigate the dominant causes of dysphagia in children in our region The second aim of this study was to estimate the prevalence of EoE in children with swallowing difficulties and to see if there were differences in the characteristics of dysphagia reported by children with EoE compared to children with non-EoE-related dysphagia.

## 2. Materials and Methods

In our retrospective analysis, we included patients with dysphagia who were referred to our Department of Pediatrics and Gastroenterology between January 2016 and December 2022. The study protocol complied with the Helsinki Accords and was approved by the local bioethics committee (approval code APK.002.244.2022). Only those children in whom dysphagia occurred before hospital admission were included in the study. They were identified by International Classification of Diseases-10 (ICD-10) diagnosis codes K20, R13.10 and R63.3. These ICD-10 codes did not change during the entire study time frame.

**Figure 1:**
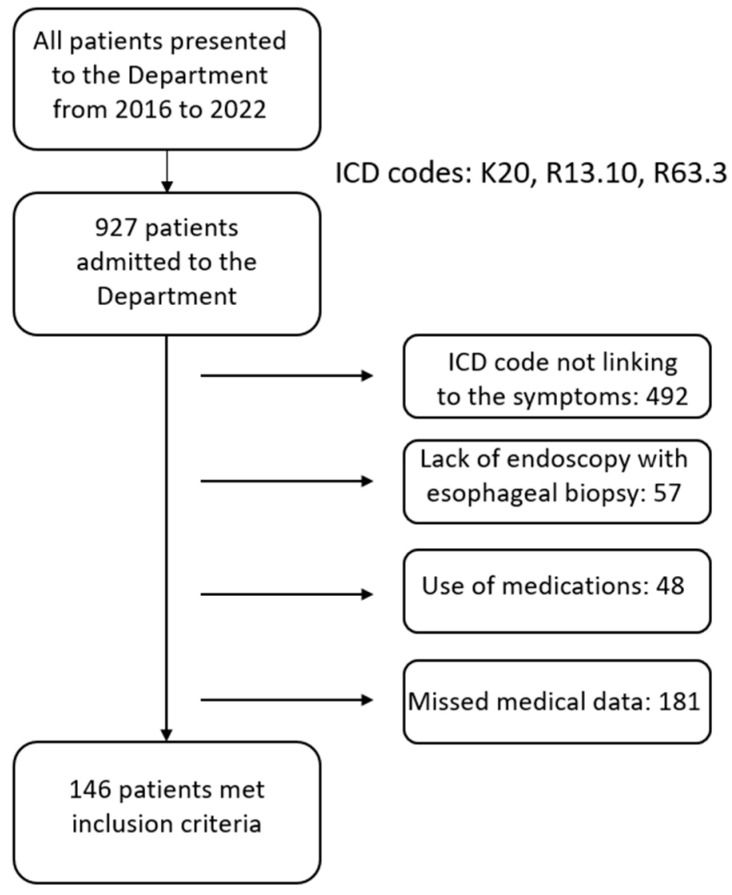
The scheme for including and excluding patients from the study.

This retrospective analysis included 924 children who were referred to our department for the reasons mentioned above. Next, the medical review was collected to confirm the presence of dysphagia. Exclusion criteria included the ICD-10 code not linking to the symptoms of the patients, failure to perform upper gastrointestinal endoscopy with esophageal biopsy, the use of medications that may affect the test result (e.g., proton pump inhibitors) and missing medical data. Patients with previously diagnosed gastrointestinal diseases that may affect the onset of dysphagia were also excluded from this study. Patients on an elimination diet were not included in our study due to its inhibitory effect on esophageal inflammation [18]. The scheme for including and excluding patients from the study is shown in Figure 1.

Symptoms considered dysphagia in infants and preschool children were described as, among others, prolonged feeding time, food refusal, nasal regurgitation, cough or choking. In older children, the identification of dysphagia was based on reported swallowing difficulties, i.e., inability to swallow, drooling, coughing or choking during swallowing. On admission to the hospital, parents/guardians filled in a standard symptom questionnaire, and then the information was completed by physicians and included in the patients’ medical records. Demographic, clinical, anthropometric, laboratory and endoscopic data were collected from medical patient records. The following causes of dysphagia were analyzed: structural anomalies of the nose, oral cavity and larynx; gastrointestinal, cardiopulmonary, neurological or psychiatric (anxiety disorders, phobias, somatic symptom disorders) and idiopathic/other disorders [3]. In all patients, body mass index (BMI) was calculated from anthropometric measurements by dividing weight (kg) by height squared (m^2^). Children were overweight or obese if their BMIs were ≥85th percentile and underweight if their BMIs were ≤5th percentile. To exclude organic causes of dysphagia, all participants underwent upper gastrointestinal endoscopy according to a standard protocol with esophageal biopsy. All participants had 24 h multichannel intraluminal impedance–pH (MII-pH) monitoring performed to diagnose gastroesophageal reflux disease (GERD). Patients were then referred for further diagnosis to otolaryngologists, speech–language pathologist and/or psychologists/psychiatrists after organic causes of dysphagia were ruled out. EoE was diagnosed according to the following criteria: symptoms of esophageal dysfunction and esophageal eosinophilia defined as at least 15 eosinophils (eos) per high power field (hpf) in histology assessment [13,19]. Patients with dysphagia were further divided into two groups: group I consisted of children with dysphagia due to EoE, while group II consisted of children with dysphagia due to other causes (non-EoE).

Statistical analysis was performed using Statistica 13.0 software. The quantitative data were expressed as median, maximum and minimum values due to nonparametric distributions. The qualitative variables were shown as absolute frequency and percentage. Comparative statistics included the chi-square test and Mann–Whitney U test. The odds ratio (OR) and 95% confidence interval (95% CI) were calculated for patient factors associated with dysphagia in the course of EoE, using multivariate binominal logistic regression. Following the rule of 10 participants per factor, power was sufficient in the multivariate regression analysis to account for 14 variables. Qualitative parameters such as sex, weight deficiency, height deficiency, allergic diseases and peripheral blood eosinophilia were analyzed. A statistical significance was noticed with *p* value < 0.05.

## 3. Results

One hundred forty-six children aged 0–17 were enrolled into this study with dysphagia as the main complaint. General details in the analyzed groups are listed in Table 1. The youngest patient with dysphagia hospitalized in our department was 8 months old, and the oldest was 17 years old; the median age of the children was 10 years. The most frequent causes of dysphagia were GI tract disorders (61.46%), followed by neurologic/psychiatric disorders (23.97%). Nearly 10% of patients had unknown etiology of dysphagia. Among the diseases of the GI tract, we noted 41.11% had EoE (n = 37/90), 22.22% exhibited inflammatory infiltrate of undetermined cause upon histologic examination of a biopsy from the esophagus (n = 20/90), 18.88% presented with gastroesophageal reflux disease (GERD) (n = 17/90), 11.11% showed ingestion of a foreign body or harmful substances (n = 10/90), 3.33% had Herpes esophagitis (n = 3/90), 1.11% exhibited Crohn diseases (n = 1/90), 1.11% presented with achalasia (n = 1/90), and 1.11% had a history of esophageal atresia (n = 1/90). Due to the exclusion of patients using PPIs, some children with GERD may not have been included in this study. This may not reflect the true prevalence of GERD in patients with dysphagia. Considering the effect of dysphagia on restricting food intake, patients were evaluated in terms of anthropometric parameters. We identified 30 underweight patients (20.55%). The most common cause of dysphagia among patients with BMI < 5 pc was neurological diseases (50%). After dividing the study group by median age (≤10 years and >10 years), it was found that gastrointestinal causes of dysphagia were significantly more common among older children (*p* = 0.005)). After dividing the study group by sex, there were no statistically significant differences in either the age of the patients or the causes of dysphagia (*p* > 0.05).

Because EoE was the most common GI cause of dysphagia, further steps in the analysis included patients with EoE compared to other causes of dysphagia unrelated to EoE (Table 2). The frequency of EoE in our study group was 25.34%. However, among the GI causes of dysphagia, EoE accounted for 41.11%. The age of EoE patients compared to non-EoE patients did not differ significantly. The youngest EoE patient was 11 months old at the time of diagnosis, while the oldest was 17 years old. Male dominance was observed in the EoE group. We did not note differences between the groups considering the duration of dysphagia and family history of GI diseases. The coexistence of allergic diseases (food allergy, asthma and allergic rhinitis) was more often reported by patients with EoE. The frequency of other symptoms reported by patients was comparable in both groups.

There was statistically significance in higher blood eosinophil count (*p* < 0.001) and eosinophilia (*p* < 0.001) in individuals with EoE in comparison to non-EoE children. To assess the effect size for categorical data, an odds ratio (OR) calculation was used, which was 29.85 for eosinophilia. The obtained result indicates an almost 30-fold higher chance of detecting eosinophilia in the group with EoE compared to the group without EoE and testifies to a large effect size. Differences in other laboratory results were insignificant (Table 3). Among the children included in the study, 40 (27.40%) had no macroscopic changes in the esophagus, including four with confirmed EoE. Among the endoscopy findings, whitish exudates, decreased vascular pattern and furrowing were the most often found lesions in patients with EoE as the typical features for EoE diagnosis. Although esophageal erosion, esophageal mucosal erythema and esophageal hernia did not meet the criterion for statistical significance, they were observed more frequently in other disorders (Table 3).

Multivariate logistic regression was used to analyze which factors might be associated with an increased risk of EoE in patients with dysphagia (Table 4). The presence of food allergy and eosinophilia had a stimulatory effect on the risk of dysphagia due to EoE.

## 4. Discussion

In this analysis, we observed that the highest prevalence of dysphagia had an organic origin from the upper GI tract, especially in older children. A significantly common cause of swallowing difficulties in the study group was EoE, the diagnosis of which requires confirmation by histological examination. It is also worth noting that in about 10% of cases we did not find the cause of dysphagia.

In our observation, the average age of onset of dysphagia was 10 years and was not statistically significantly different when patients were divided by sex. A similar median age result of 8.2 years was obtained by Bhattacharyya et al. [3]. However, in other studies, children with dysphagia were younger than in our observations (from 6.6 months to 1.14 years) [20,21]. Considering the causes of dysphagia, some are more specific to selected age groups. In our study, the predominance of GI diseases was observed among older children. To the best of our knowledge, studies published to date have focused on selected age groups or have not analyzed the causes of dysphagia according to the age of patients [3,20,21,22,23]. Therefore, future studies on a larger group of children are needed.

Difficulty in swallowing is one of the most common symptoms of EoE in adults and adolescents. The incidence of dysphagia in children with EoE varied between studies and ranged between 22% and 44% [16,24]. We found that about a quarter of children with dysphagia had EoE. The pathophysiology of dysphagia in EoE is still not fully understood. Symptoms of dysphagia may be associated with anatomical distortions as well as dysmotility [25]. Dysmotility in EoE arises from fibrosis of a deeper muscle layer and is an effect of an inflammatory process fixed by eosinophils and mast cells [26]. Routine use of immunohistochemistry would perhaps help to gain a deeper understanding of the mechanism of dysphagia in EoE [27]. In our study, EoE was the predominant cause of dysphagia. To the best of our knowledge, no studies evaluating the prevalence of EoE among pediatric patients with dysphagia have yet been published. Svystun et al. characterized a cohort of 128 children with dysphagia and found 3 cases of esophagitis of an idiopathic cause [20]. Similarly, Lefton-Greif et al., in a group of 19 children with dysphagia manifested by unexplained respiratory problems, found idiopathic esophagitis in 1 child [22].

In our observations, there were no coexisting features of dysphagia that could help in the diagnostic study; only the coexistence of allergy and peripheral eosinophilia could indicate EoE. Atopic diseases are more frequently observed in EoE patients in comparison to the general population [13]. In our study, children with dysphagia in the course of EoE had higher incidence of food allergy, allergic rhinitis and asthma. In addition, the presence of food allergy was one of the risk factors for the diagnosis of EoE in children hospitalized for dysphagia.

In adult patients with dysphagia and EoE, the incidence of allergic diseases was 50%. It is worth noting that this study did not evaluate the statistical significance of the incidence of allergies compared to non-EoE patients [28]. However, in another analysis of patients with dysphagia, the incidence of allergic diseases was comparable in the EoE and non-EoE groups [29]. Considering the results of laboratory tests in our study, patients with EoE had significantly higher absolute serum eosinophil counts, which is consistent with other observations [16,30,31]. Furthermore, in our observation, eosinophilia was the risk factor for EoE among children with dysphagia. Our results suggest that special attention should be paid to children with symptoms of dysphagia and coexisting food allergy and peripheral eosinophilia, as they have a higher risk of EoE diagnosis.

The diagnosis of dysphagia is based on invasive tests. MII-pH and esophageal manometry allow the diagnosis of GERD, esophageal motor and functional disorders. In our study group, GERD was confirmed in 11.64% of children with dysphagia, and achalasia was confirmed in less than 1%. In other studies, GERD was the cause of dysphagia in 28% of adults [32]. Population-based studies on the prevalence of dysphagia in children with GERD have not yet been conducted. However, Fishbein et al. observed a higher prevalence of dysphagia among children with GERD like symptoms (unconfirmed in MII-pH) compared to controls [33]. Another diagnostic procedure is endoscopic and histological examination of esophageal biopsy specimens to detect organic and anatomical changes of the esophagus as the cause of dysphagia. On the other hand, not all histological findings are specific to the diagnosis. In our study, despite an abnormal histological result, a diagnosis could not be made in 22.22% of cases. The endoscopic diagnosis of EoE is based on the presence of typical lesions such as edema, rings, exudates, fissure, stricture and an infiltration of eosinophilia in the esophageal mucosa evaluated by histological examination. Rieker et al. noticed that 80% of patients with dysphagia in the course of EoE have features of esophageal rings, furrows or a combination of both in endoscopy [28]. Gunasekaran et al. observed that children and adolescents, depending on the dominant symptoms of EoE (dysphagia vs. abdominal pain), differed in the visual endoscopy findings of the esophagus. For example, linear furrowing was observed more frequently in patients with dysphagia, which was consistent with our observations [34]. We showed that a whitish exudate, reduced vascular pattern and furrowing were associated with a histologically confirmed diagnosis of EoE. Moreover, trachealization was observed only among cases of EoE. In contrast, other macroscopic changes of the esophagus (stenosis, mucosal edema, esophageal erosion, esophageal mucosal erythema) did not differentiate between patients with EoE and other conditions. Despite macroscopic changes suggesting the diagnosis of EoE, a definitive diagnosis can only be made based upon finding eosinophilic infiltrates in the esophageal mucosa. Interestingly, among four patients with EoE, the macroscopic picture of the esophagus was normal. If EoE is clinically suspected, several esophageal biopsies should be taken despite a normal esophageal picture on endoscopy [19].

The main limitation of the study is the retrospective design of the study. Another limitation is the small number of patients included in the study. This was influenced by the coronavirus disease 2019 (COVID-19) pandemic and the associated reduction in hospital admissions. It is worth mentioning that some patients with other conditions (e.g., cerebral palsy) may have been hospitalized in other departments and were therefore not included in our analysis. The strength of our study lies in the thorough characterization of demographic, clinical and endoscopic data in children with dysphagia. To the best of our knowledge, this is the first report on the incidence and predictive factors of EoE among children with dysphagia.

## 5. Conclusions

We showed that most cases of dysphagia were related to gastrointestinal disorders in pediatric patients admitted to the department. EoE was common cause of dysphagia in children. Patients with dysphagia, comorbid allergy and peripheral blood eosinophilia should be suspected for having EoE and referred for endoscopy. Further studies including larger cohorts of patients with a follow-up period are needed to investigate the course of dysphagia, especially in EoE children and adolescents.

## Figures and Tables

**Table 1:** Characteristics of patients with dysphagia.

Number of patients	146
Age (years) (median, range)	10 (0–17)
Male (n, %)	81 (55.48%)
Duration of dysphagia (days) (median, range)	60 (1–1500)
BMI:	
<5 percentile (n, %)	30 (20.55%)
>85 percentile (n, %)	0 (0.0%)
Short stature (<3 percentile) (n, %)	18 (12.33%)
Cause of dysphagia:	
Structural anomalies of nose, oral cavity and larynx (n, %)	5 (3.42%)
Gastrointestinal tract disorders (n, %)	90 (61.64%)
Cardiopulmonary disorders (n, %)	1 (0.68%)
Prematurity (n, %)	1 (0.68%)
Neurological/psychiatric disorders (n, %)	35 (23.97%)
Idiopathic (n, %)	14 (9.59%)

BMI—body mass index; n—number of cases.

**Table 2:** Comparative characteristics of patients with EoE and non-EoE.

	EoE (n = 37)	Non-EoE (n = 109)	*p*
Age (years) (median, range)	10 (0–17)	10 (0–17)	0.40
Male (n, %)	28 (75.68%)	53 (48.62%)	0.004
Duration of dysphagia (days) (median, range)	60 (1–60)	48 (2–1500)	0.63
Family history of gastrointestinal diseases (n, %)	2 (5.41%)	4 (3.67%)	0.65
Comorbidities:			
Food allergy (n, %)	16 (43.24%)	8 (7.34%)	<0.001
Asthma	8 (21.62%)	2 (1.83%)	<0.001
Allergic rhinitis	13 (35.14%)	6 (5.50%)	<0.001
Atopic dermatitis	2 (5.41%)	2 (1.83%)	0.25
Symptoms:			
Abdominal pain	13 (35.14%)	37 (33.94%)	0.90
Halitosis (n)	3 (8.11%)	5 (4.59%)	0.42
Vomiting (n)	7 (18.92%)	24 (22.02%)	0.69
Lack of appetite (n)	2 (5.41%)	12 (11.01%)	0.32
Heartburn (n)	2 (5.41%)	10 (9.17%)	0.47
Nausea (n)	2 (5.41%)	11 (10.09%)	0.46
Weight loss (n)	5 (13.51%)	17 (15.60%)	0.76
Eructation (n)	0 (0.00%)	7 (6.42%)	NA
Regurgitation (n)	3 (8.11%)	5 (4.59%)	0.42
BMI < 5 percentile (n, %)	8 (21.62%)	22 (20.18%)	0.85
Short stature (<3 percentile) (n, %)	6 (16.22%)	12 (11.00%)	0.41

BMI—body mass index; n—number of cases; NA—not applicable.

**Table 3:** Laboratory results and endoscopy findings in EoE and non-EoE groups.

	EoE (n = 37)	Non-EoE (n = 109)	*p*
Laboratory result:			
Blood eos count	610 (10–2560)	130 (0–2643)	<0.001
Eosinophilia (n) (>400 eos/µL)	26 (70.27%)	8 (7.34%)	<0.001
Hb (median, min-max) (g/dL)	13.3 (11.5–17.0)	12.8 (10.6–17.0)	0.68
CRP (median, min-max) (mg/dL)	0.6 (0.08–4.90)	0.6 (0.05–91.52)	0.07
PLTs (median, min-max) (×10^3^/µL)	287 (159–474)	282 (137–624)	0.99
Endoscopic findings in esophagus:			
Whitish exudates (n, %)	10 (27.03%)	4 (3.67%)	<0.001
Stricture (n, %)	4 (10.81%)	3 (2.75)	0.05
Decrease vascular pattern (n,%)	23 (62.16%)	16 (14.68)	<0.001
Furrowing (n,%)	32 (86.49%)	8 (7.34)	<0.001
Mucosal edema (n,%)	5 (13.51%)	6 (5.50%)	0.12
Erosion (n,%)	6 (16.22%)	33 (30.28%)	0.09
Mucosal erythema (n,%)	1 (2.70%)	6 (5.50%)	0.49
Papules/plaques (n,%)	3 (8.11%)	4 (3.67%)	0.27
Trachealization/rings (n,%)	10 (27.03%)	0 (0.0%)	NA
Esophageal hernia (n,%)	0 (0.0%)	2 (1.83%)	NA
Histology examination:			
Median eos counts/hpf (range)	20 (15–45)	0 (0–10)	NA

CRP—C-reactive protein; eos—eosinophil; Hb—hemoglobin; hpf—high power field; n—number of cases; PLTs—platelets.

**Table 4:** Factors associated with diagnosis of EoE in patients with dysphagia using multivariate binominal logistic regression modeling.

	Multivariate OR (%CI)	*p*
Male (n, %)	1.90 (0.54–6.73)	0.32
BMI < 5 percentile (n, %)	0.58 (0.11–3.25)	0.54
Short stature (<3 percentile) (n, %)	1.93 (0.26–14.54)	0.52
Food allergy (n, %)	7.46 (1.58–35.15)	0.01
Asthma (n, %)	7.97 (0.712–89.22)	0.09
Allergic rhinitis (n, %)	2.95 (0.61–14.17)	0.18
Atopic dermatitis (n, %)	0.76 (0.03–19.71)	0.87
Eosinophilia (n) (>400 eos/µL)	27.19 (7.60–97.25)	<0.001

BMI—body mass index; eos—eosinophils; n—number of cases.

## Data Availability

The datasets generated and analyzed during the current study are available from the corresponding author on reasonable request.

## Abbreviations

The following abbreviations are used in this manuscript:

EoE	Eosinophilic esophagitis
ICD-10	International Classification of Diseases-10
MII-pH	Multichannel intraluminal impedance-pH
GERD	Gastroesophageal reflux disease
Eos	Eosinophils
hpf	High power field
OR	Odds ratio
CI	Confidence interval
GI	Gastrointestinal
BMI	Body mass index
COVID-19	Coronavirus disease 2019
